# Linguistic validation of a widely used recovery score: quality of recovery-15 (QoR-15)

**DOI:** 10.3906/sag-2108-41

**Published:** 2021-12-18

**Authors:** Umut KARA, Fatih ŞİMŞEK, Hasan KAMBUROĞLU, Mehmet Özgür ÖZHAN, Ümit ALAKUŞ, Mehmet Emin İNCE, Sami EKSERT, Gökhan ÖZKAN, Mehmet Burak EŞKİN, Serkan ŞENKAL

**Affiliations:** 1 Department of Anesthesiology and Reanimation, Gülhane Training and Research Hospital, University of Health Sciences, Ankara, Turkey; 2 Department of Anesthesiology and Reanimation, Tuzla State Hospital, İstanbul, Turkey; 3 Department of Anesthesiology and Reanimation, Private Çankaya Hospital, Ankara, Turkey; 4 Department of General Surgery, Gülhane Training and Research Hospital, University of Health Sciences, Ankara, Turkey

**Keywords:** Patient reported measures, quality of recovery, cross cultural comparison

## Abstract

**Background/aim:**

The quality of recovery-15 (QoR-15) is a patient reported outcome questionnaire that measures the quality of recovery after surgery and anesthesia. The QoR-15 has been validated in many languages; Turkish version of the QoR-15 has not yet been established. The aims of this study were to translate the QoR-15 questionnaire into Turkish and to perform a full psychometric evaluation of the Turkish version.

**Materials and methods:**

After translating the original English version of the QoR-15 scale into Turkish, the QoR-15T scale was psychometrically validated. This process included validity, reliability, responsiveness, feasibility. The QoR-15T was evaluated before the surgery and 24 h after surgery.

**Results:**

A total of 210 patients completed the pre- and postoperative questionnaires, providing a completion rate of 93.75%. The correlation coefficient between QoR-15T score and VAS score was 0.644 on postoperative day 1 (p < 0.001). Inter item Cronbach’s alpha was 0.863. Global test-retest concordance coefficient was 0.98 (95% CI: 0.94–1.00).

**Conclusion:**

The QoR-15T scale is a reliable and valid instrument for evaluating postoperative quality of recovery in Turkish speaking patients. The psychometric characteristics used to assess postoperative quality of recovery were similar to those in the English version.

## 1. Introduction

Recovery is a multidimensional and complex process influenced by a variety of factors such as patient characteristics, surgical procedures, and anesthesia [1]. The great majority of research into recovery from surgery and anesthesia has mostly focused on physiological parameters including pain, nausea/vomiting, recovery of bowel function, length of hospital stay, recovery timeframes, and the occurrence of adverse events such as poor outcomes and mortality [2,3]. There is an increasing focus on patient-perceived quality of recovery (QoR). Patient reported outcome measures (PROMs) can be used to assess the patient’s perspective.

Myles et al. developed the quality of recovery - 40 score (QoR - 40) in 2000, and it is now widely used [4]. It has also been successfully translated and validated in the Turkish language [5]. In 2013, the quality of recovery-15 score (QoR-15) was derived from the QoR - 40 score [6]. The QoR-15 score has been validated and demonstrated to perform as well as the QoR - 40 score. The QoR-15 scale is a unidimensional measurement of quality of recovery measured in five domains: physical comfort, pain, physical independence, psychological support, and emotional state. The QoR-15 scale provides a score ranging from 0 to 150, with a high score indicating a good quality of recovery. It has been validated in various linguistic and cultural contexts, including Japanese [7], Italian [8], French [9], Korean [10], Chinese [11], Portuguese [12], Danish [13], and Swedish [14]. All the translated versions of QoR-15 show adequate validity and reliability for evaluating recovery quality. There is no validated translation of the short-form version in the Turkish language.

The aim of this study was to develop the Turkish version of QoR-15 (QoR-15T) by a translation and cultural adaptation process and to evaluate the validity, reliability, and responsiveness of the QoR-15T for Turkish patients who receive general anesthesia. The authors hypothesized that the QoR-15T would have as comprehensive validity, reliability, and responsiveness as the original English version.

## 2. Materials and methods

### 2.1. Patient selection

This prospective observational cohort study was approved by the Ethics Committee of Gülhane Training and Research Hospital, Turkey (No. 2020 / 246) and was registered with ClinicalTrials.gov (NCT04726605, January 27, 2021). The study was conducted in accordance with the principles of the Declaration of Helsinki [15]. All procedures were applied following the Strengthening the Reporting of Observational Studies in Epidemiology guideline [16]. Patients undergoing surgery at the hospital, between January 2021 and July 2021, were enrolled.

The inclusion criteria were age > 18 years, American Society of Anesthesiologists (ASA) physical status (PS) ≤ III, being able to read and speak in Turkish, and to be scheduled for general, thoracic, plastic, gynecological, urological, ophthalmic, orthopedic surgery, or neurosurgery under elective general anesthesia. The study exclusion criteria were defined as not being fluent in the Turkish language, being unable to provide written informed consent, the presence of a neuropsychiatric disorder that might bias QoR-15T measurements, or the application of emergency surgery. Patients who were not expected to be able to answer the QoR-15T scale on postoperative day 1 were also excluded.

### 2.2. Development of the QoR-15T

**Figure 1:**
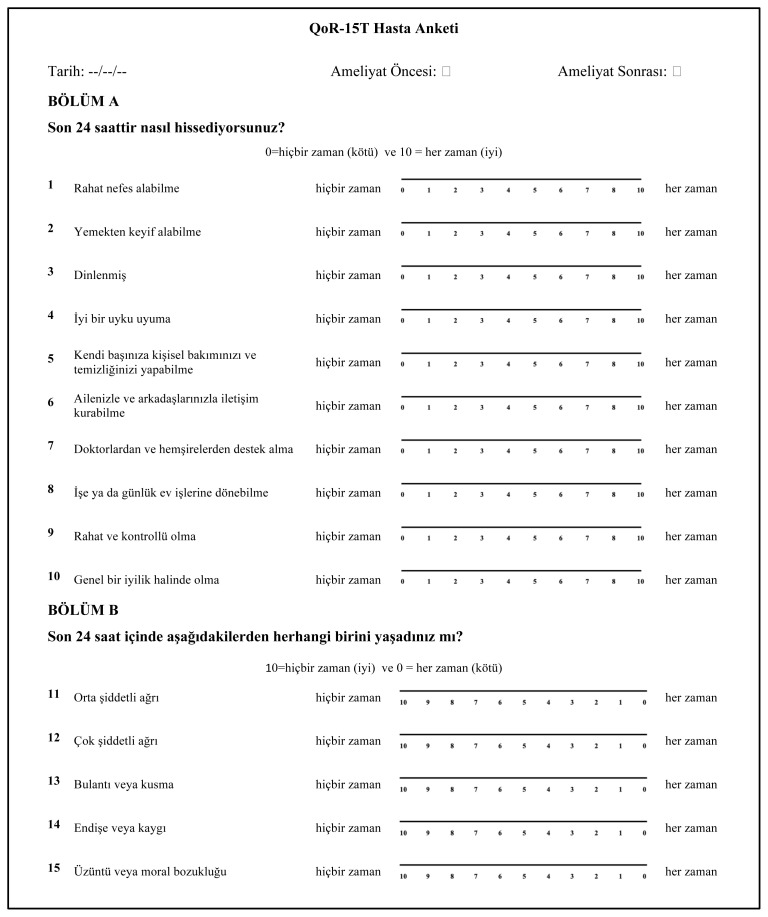
QoR-15T survey in Turkish.

Permission was received from the author (Myles PS) of the original English language version of the QoR-15 scale. The translation technique was performed in accordance with the recommendations of Beaton and Bullinger [17]. First, two authors (coauthors UK and MEI) translated the QoR-15 into Turkish with reference to the Turkish version of the QoR - 40 (QoR – 40T), which has been validated [5]. A temporary Turkish version of the QoR-15 was agreed upon, which was then back-translated by a third person (co-author SŞ: healthcare experience in USA and Turkey). A consensus was reached regarding the Turkish version of the QoR-15, and this was then tested on a randomly selected cohort of 10 postoperative anesthesia care nurses. All QoR-15T questions were confirmed to be comprehensible. As a result, the final Turkish version of the QoR-15 (QoR-15T) was created (Figure 1).

### 2.3. Data collection

Demographic characteristics were recorded preoperatively. Intraoperative data were obtained from electronic and print patient records. The level of education was classified as elementary, high school, and university. The patients were separated into two groups as minor-intermediate and major-complex, depending on the extent of the surgery. This classification of surgical operations was made according to the SORT classification (Surgical Outcome Risk Tool) [18]. The time between the start and the end of the surgery was recorded as the duration of surgery (min.), and the time between arrival and exit from the post-anesthesia care unit (PACU) was defined as the PACU time (min.).

On the morning of the surgery day, the QoR-15T scale was presented to the patient. The QoR-15T was applied both preoperatively (operating room waiting area) and at the postoperative 24th h (ward). At the 25th h, a random subset of 20 patients were requested to complete the QoR-15T scale once again. The time required to complete each QoR-15T scale was recorded. Together with the QoR-15T questionnaire, the 100 mm global QoR visual analog scale (VAS) was used to assess overall well-being. The VAS scale ranges from 0 to 100 mm, indicating poor to excellent recovery. The QoR-15T scale and the VAS scale were administered using self-assessment with assistance as required.

### 2.4. Psychometric evaluation of the QoR-15T

For convergent validity, the QoR-15T score was correlated to the 100 mm global QoR VAS assessment of global health status. Interdimensional and interitem correlations for the QoR-15T were measured.

To measure construct validity, the correlation of global QoR-15T score and subdimension scores with continuous parameters were assessed. The global QoR-15T score and subdimension scores were compared according to sex, ASA PS, smoking, alcohol consumption, education level, and extent of surgery. Discriminant validity was tested by comparing the QoR-15T score in two groups according to the VAS (≥ 70 mm [good] vs. < 70 mm [poor]) [6].

Reliability was measured for consistency of the QoR-15T. Reliability was assessed by internal consistency (cronbach’s alpha), split-half reliability, and test-retest reliability. The latter was tested between two repeated measurements in a convenience sample of 20 patients 60 min after the postoperative QoR-15T test. The floor and ceiling effect were determined by calculating if 15% of respondents had either the highest (150) or lowest possible score (0).

Standardized response mean (SRM) and Cohen’s effect size were used to assess responsiveness. The SRM was calculated as the change scores divided by the SD of the change scores. The Cohen effect size was calculated as the average change scores (from preoperative to postoperative) divided by the SD at baseline. The patient recruitment rate and successful completion rate were used to assess the clinical feasibility of the QoR-15T.

### 2.5. Statistical analysis

There is no standard approach to determine the sample size in scale validity and reliability studies. It is typically recommended that the minimum number of participants is 150 or that the scale be administered to 10–20 times the number of items on the scale [19,20]. To analyze 225 patients in the QoR-15T validation study, 250 patients were to be included in the study to account for potential data loss and other possible causes.

The measurement data were presented as mean ± SD values and categorical data as number (n) and percentage (%). The normal distribution of the continuous variables was tested using the Kolmogorov–Smirnov test. Associations were measured using Spearman rank (ρ) correlation coefficient. Internal consistency was measured using Cronbach α. Test-retest reliability was measured using the intraclass correlation coefficient. Changes from baseline were compared using the signed ranks test. The student’s t-test was used to compare data with normal distribution, and the Mann–Whitney U-test was applied to data not showing normal distribution, and the Chi-square test was used to compare categorical data. All statistical analyses were performed using SPSS version 25.0 for Windows software (IBM Corp. Released 2017. IBM SPSS Statistics for Windows, Version 25.0. Armonk, NY: IBM Corp.). The null-hypothesis was rejected if two-tailed p < 0.05.

## 3. Results

**Figure 2:**
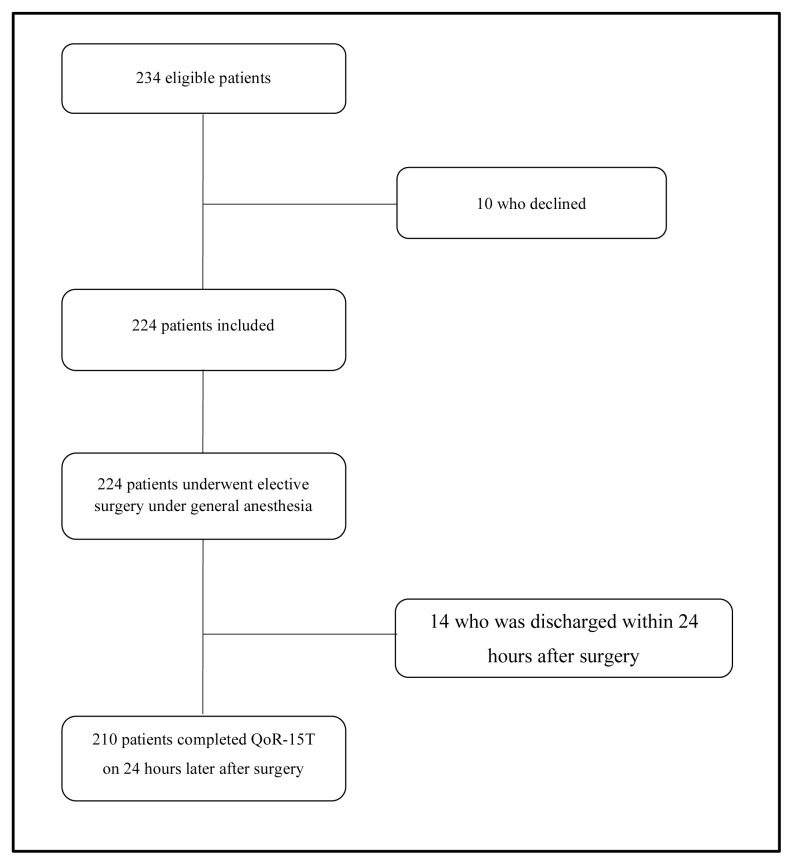
Study flow chart.

Of the 234 eligible patients, 10 refused to participate in this study, resulting in a 95.7% (224 / 234) recruitment rate. Fourteen patients were discharged within 24 h after surgery; thus, the completion rate at 24 h after surgery was 93.8% (210 / 224) (Figure 2). The mean time taken to complete the postoperative QoR-15T scale was 2.9 ± 0.7 min (range: 2–8 min).

To assess for convergent validity, the correlation was evaluated between the QoR-15T and the VAS for recovery. The Spearman rho (ρ) correlation coefficient was 0.644 on the postoperative first day following surgery (p < 0.001). A strong correlation was determined between QoR-15T and VAS (correlation > 0.60). The correlation of each domain of QoR-15T with VAS was calculated. The VAS score was determined to be weakly correlated with psychological support (correlation < 0.30) and moderately correlated with other domains (correlation 0.30–0.60) (p < 0.001). The correlations of postoperative VAS and each domain of the QoR-15T are shown in Table 2.

A negative, moderate (ρ = −0.410), statistically significant correlation was determined between QoR-15T and surgical time (p < 0.001). A negative, moderate (ρ = −0.380), statistically significant correlation was determined between QoR-15T and PACU length of stay (p < 0.001). The QoR-15T and subscale scores were compared according to sex, ASA physical status, education level, and extent of surgery. A statistically significant difference between the scores of males and females was only seen in the subscale of psychological support (17.65 ± 3.62 vs. 18.84 ± 2.06, p = 0.042). In terms of ASA physical status, a statistically significant difference was seen only in the subscale of psychological independence (p = 0.04). No statistically significant differences were seen between the variables of education levels. There was a statistically significant difference between minor-intermediate surgery and major-complex surgery in respect of the global QoR-15T scores (120.62 ± 21.06 vs. 108.26 ± 24.35, p = 0.006). In the variable of grade of surgery, a statistically significant difference was observed between the minor-intermediate and major-complex scores in the psychological support subscale (19.04 ± 2.18 vs. 17.52 ± 3.42, p = 0.009) and in the psychological independence subscale (13.47 ± 5.28 vs 9.89 ± 6.79, p = 0.003).

Patients with a good or poor postoperative recovery, as indicated by a VAS value of ≥ 70 or < 70 mm, were compared to establish discriminant validity. The QoR-15T score was significantly different between these groups (73–149; IQR = 22) and (44–134; IQR = 34), (p < 0.0001).

Reliability was evaluated by Cronbach α for internal consistency. Inter item Cronbach α was 0.863, and interdimension Cronbach α was 0.755. The inter item and inter dimension correlation matrices for QoR-15T at 24 h postoperatively are shown in Tables 3 and 4. The Cronbach α values for global QoR-15T and its dimensions in the preoperative and postoperative period are shown in Table 5. The median item to own dimension, the Cronbach α, and coefficients for each dimension were physical comfort (α = 0.688, ρ = 0.861), emotional state (α = 0.648, ρ = 0.825), psychological support (α = 0.748, ρ = 0.599), physical independence (α = 0.704, ρ = 0.722), and pain (α = 0.741, ρ = 0.600). The split half coefficient was 0.78, global test-retest concordance coefficient was 0.98 (95% CI: 0.94–1.00) for the total QoR-15T score. The proportion of patients achieving the highest possible QoR-15T score at 24 h was 0% (n = 0 / 210), and the proportion of patients achieving the lowest score was 0% (n = 0 / 210), and, therefore, neither floor nor ceiling effects of the scoring instrument were demonstrated.

Changes in the preoperative and postoperative QoR-15T and responsiveness are presented in Table 6. The postoperative QoR-15T score decreased to 114.50 ± 23.47 from the preoperative QoR-15T score of 127.36 ± 17.53 (p < 0.001). The preoperative and postoperative scores showed significant differences. The Cohen’s effect size and SRM for preoperative and postoperative QoR-15T were 0.59 and 0.57, respectively.

## 4. Discussion

This study was conducted to assess the psychometric properties of the QoR-15T and to compare these with the VAS values in adult patients undergoing elective surgery under general anesthesia. The study results revealed that QoR-15T is a valid, reliable, responsive, and easy to use instrument for the assessment of postoperative recovery in the Turkish population.

To assess convergent validity, the QoR-15T for recovery was compared with the VAS values. Convergent validity showed that the correlation coefficient between the QoR-15T score and VAS exceeded the published recommendation (ρ > 0.60), which was similar to the coefficient in the original study [6]. There is no gold standard for the assessment of the quality of postoperative recovery. Although VAS is a simple assessment instrument with no subdimensions, it was used in this study because it was used in the original QoR-15 article [6].

There was an inverse correlation between QoR-15T and surgery time and PACU time. These correlations were weaker than those mentioned in the original study [6]. In other similar studies including QoR-15 validation, the surgery time and PACU time were likewise inversely connected with global QoR-15 scores [9,13,14]. It was also seen that patients with a higher ASA PS had lower QoR-15T scores.

The discriminate validity of the QoR-15T was determined by comparing the scores of patients who had minor and intermediate surgery to those who had major and complex surgery, with the latter scoring significantly lower. Patients with a good or poor postoperative recovery, as determined by the VAS score, were compared to demonstrate discriminant validity. The VAS score was used to classify good and poor recovery, as it is a more objective assessment than the clinician’s or patient’s opinion [6,9,10].

Cronbach alpha and split-half reliability were used to analyze internal consistency. These two coefficients were both > 0.7, which satisfied the published recommendations. The QoR-40T scale inter item correlation coefficients ranged from 0.40 to 0.76, with item 7 having the lowest value. Although the original scale included groups of negatively correlated binary items, the QoR-15T scale did not have negative correlated binary items. The interdimensional correlation coefficients ranged from 0.59 to 0.86, with psychological support scoring the lowest and physical comfort scoring the highest. There was no negative correlation between binary subdimensions, or between binary items. These data were sufficient to conclude that the QoR-15T has adequate reliability.

The Cronbach alpha value of the QoR-40T scale (0.936) is higher than the Cronbach alpha value of the QoR-15T scale (0.863). Both are higher than 0.7, indicating that QoR-15T can be used in place of QoR-40T to achieve the same reliability in a shorter time. As a result of the reliability analysis of the QoR-15T scale, as the corrected item-total correlations of all the items were greater than 0.3 (range: 0.355 to 0.714), it can be concluded that all the items adequately contribute to reliability. The results of test-retest reliability of the QoR-15T indicated that QoR-15T has an acceptable level of reliability. For the test-retest, the value of Cronbach α was 0.98, which exceeds the established criterion of 0.7 for good reliability.

Cohen effect size and SRM values > 0.5 suggest a moderate intervention impact, and values > 0.8 suggest a strong intervention impact [21,22]. In the difference between the preoperative and postoperative measurements in the QoR-15T scores, the Cohen effect size (0.59) and SRM (0.57) demonstrated a moderate effect with the results. Although the QoR-15T scale had a moderate effect with these values, they were lower than the original QoR-15 scale effect size (1.35) and SRM (1.04) levels. Similarly, the Italian QoR-15 validation study revealed comparable SRM results (0.64) [8]. The SRM value of the QoR-15T scale had an impact that was quite similar to the SRM value of the QoR-40T scale (0.62) [5]. The subdimensions of the Turkish scale were used to evaluate Cohen effect size and SRM, while the original scale was based on items. Although the emotional state subdimension score increased over the postoperative period, it was not statistically significant, and all other subdimension scores declined. Possible explanations for the difference in subdimensions between organizations could include cultural variations and the timing of the pre-postoperative scale use.

The acceptability and feasibility of the QoR-15T was assessed using recruitment rate, completion rate, and time taken to complete the questionnaire. There was a high rate of participation and successful completion, and most patients were able to complete the questionnaire in less than 4 min. Since the completion times for the QoR-40T scale are not specified, it cannot be said what improvement the QoR-15T scale brings in terms of time. Less than 4 min has been shown to be a reasonable time to measure the quality of the review in clinical studies.

During the implementation of the QoR-15T scale, certain difficulties were observed. When moving from question 10 to question 11, the inversion of the Likert scale caused confusion.

It was also observed that, as the patient transitioned from part A to part B, the practitioner had to assist the patient by stating 0 for “worst possible scenario” and 10 for “best possible scenario”. As previously stated, this was the most negative aspect of the QoR-15T scale. In addition, the fact that there are two questions (items 11 and 12) on the scale to assess pain can create difficulty in understanding. In the current study, patients referred to “tolerable pain” for moderate pain and “terrible pain” for severe agonizing pain.

There were several limitations in this study, since it was conducted in a single, tertiary level hospital in Turkey. The first limitation was that, as the study included patients undergoing elective surgeries under general anesthesia, caution was required when applying the QoR-15T to emergency surgery. Second, patients undergoing outpatient surgery, cardiac, and otolaryngological surgery were not included. Finally, patients with poor understanding of Turkish and serious preexisting medical conditions were excluded.

In conclusion, the QoR-15 was translated into Turkish, and its usefulness was assessed in surgical patients who underwent various surgeries under general anesthesia. The results demonstrated that the QoR-15T is a valid, reliable, feasible, and responsive method of assessing postoperative recovery. When compared to the QoR-40T, the QoR-15T provides an equally comprehensive but less time-consuming assessment of a patient’s QoR following anesthesia and surgery. These results support the use of the QoR-15T during the perioperative period to evaluate patient recovery after anesthesia and surgery.

## Figures and Tables

**Table 1:** Patient demographic and surgical characteristics (n = 230).

	Value
Age (years)	48.07 ± 14.17
Sex	
Male	106 (46%)
Female	124 (54%)
ASA physical status score	
1	43 (18%)
2	164 (71%)
3	23 (11%)
Weight (kg)	75.61 ± 14.09
Height (cm)	167.04 ± 8.30
BMI (kg/m^2^)	26.96 ± 4.88
Smoking status	
Nonsmoker	138 (60%)
Current smoker	92 (40%)
Drinking status	
Nondrinker	216 (94%)
Drinker	14 (6%)
Education (n)	
Elementary	94 (40.9%)
High school	68 (29.6%)
University	68 (29.6%)
Extent of surgery	
Minor - Intermediate	120 (52.2%)
Major - Complex	110 (47.8%)
Type of surgery (n)	
General	87 (38%)
Neurosurgical	16 (7%)
Thoracic	11 (5%)
Plastic and Reconstructive	20 (9%)
Gynecologic	21 (9%)
Urologic	21 (9%)
Ophtalmic	18 (8%)
Orthopedic	36 (15%)
Duration of surgery (min.)	133.82 ± 77.27
PACU time (min.)	33.39 ± 11.68

**Table 2:** Correlation between the global QoR VAS and the QoR-15T (Spearmann’s ρ).

	Postoperative VAS	p value
QoR-15T	0.644	< 0.001
Physical Comfort	0.577	< 0.001
Emotional State	0.589	< 0.001
Psychological support	0.264	0.006
Physical Independence	0.459	< 0.001
Pain	0.461	< 0.001

Correlation is significant at the 0.01 level (2-tailed).

**Table 3:** Interitem correlation for the postoperative QoR-15T score. Bold values are statistically significant values.

	Total score	1	2	3	4	5	6	7	8	9	10	11	12	13	14
1. Able to breathe easy	**0.564** **	**-**													
2. Been able to enjoy food	**0.680** **	**0.376** **	**-**												
3. Feeling rested	**0.768** **	**0.586** **	**0.589** **	**-**											
4. Have had a good sleep	**0.688** **	**0.452** **	**0.520** **	**0.718** **	**-**										
5. Able to look after personal toilet and hygiene unaided	**0.696** **	**0.301** **	**0.496** **	**0.511** **	**0.374** **	**-**									
6. Able to communicate with family or friends	**0.568** **	**0.303** **	**0.519** **	**0.465** **	**0.311** **	**0.505** **	**-**								
7. Getting support from hospital doctors and nurse	**0.402** **	**0.325** **	0.129	**0.261** *	**0.248** **	**0.172** *	**0.398** **	**-**							
8. Able to return to work or usual home activities	**0.577** **	0.068**	**0.346** **	**0.355** **	**0.392** **	**0.527** **	0.148	0.011	**-**						
9. Feeling comfortable and in control	**0.750** **	**0.396** **	**0.354** **	**0.592** **	**0.474** **	**0.409** **	**0.580** **	**0.499** **	**0.346** **	**-**					
10. Having a feeling of general well-being	**0.710** **	**0.404** **	**0.365** **	**0.589** **	**0.527** **	**0.343** **	**0.377** **	**0.422** **	**0.375** **	**0.730** **	**-**				
11. Moderate pain	**0.467** **	0.193	**0.188** *	**0.246** **	**0.218** **	**0.196** **	0.000	0.119	0.167	**0.376** **	**0.375** **	**-**			
12. Severe pain	**0.564** **	**0.245** *	0.174	**0.201** *	**0.241** **	**0.296** **	0.136	**0.264** **	**0.257** **	**0.413** **	**0.381** **	**0.470** **	**-**		
13. Nausea or vomiting	**0.447** **	**0.371** **	**0.282** **	**0.263** **	0.134	0.290	**0.101** *	**0.062** *	**0.144** *	**0.157** **	0.104	**0.150** *	**0.328** **	**-**	
14. Feeling worried or anxious	**0.511** **	**0.186** *	**0.226** **	**0.225** *	**0.233** *	**0.208** *	**0.215** **	**0.291** **	0.205	**0.340** **	**0.270** **	**0.234** **	**0.248** **	**0.336** **	**-**
15. Feeling sad or depressed	**0.530** **	**0.239** **	**0.213** *	**0.250** *	**0.232** **	**0.222** *	**0.186** **	**0.222** **	0.158	**0.308** **	**0.332** **	**0.227** **	**0.493** **	**0.311** *	**0.634** **

** Correlation is significant at the 0.01 level (2-tailed).

* Correlation is significant at the 0.05 level (2-tailed).

**Table 4:** Interdimensional correlation for the QoR-15T. Bold values are statistically significant values.

	Total Score	Physical Comfort	Emotional State	Psychological Support	Physical Independence	Pain
Physical Comfort	**0.861** **	-				
Emotional State	**0.825** **	0.572	-			
Psychological support	**0.599** **	0.480	0.526	-		
Physical Independence	**0.722** **	**0.521** **	**0.426** **	**0.328** **	-	
Pain	**0.600** **	**0.339** **	**0.526** **	0.133	**0.302** **	-

** Correlation is significant at the 0.01 level (2-tailed).

**Table 5:** Cronbach α for preoperative and postoperative QoR-15T.

	Cronbach α (preoperative)	Cronbach α (postoperative)
Global QoR-15T	0.807	0.863
Physical Comfort	0.528	0.688
Emotional State	0.525	0.648
Psychological support	0.673	0.748
Physical Independence	0.606	0.704
Pain	0.656	0.741

**Table 6:** Change in QoR-15T of patients the day before surgery (preoperative) and the first day f following surgery (postoperative).

	Max score	Preoperative	Postoperative	% Change from baseline	p value	Cohen’s Effect size	SRM
Global QoR-15T	150	127.36 ± 17.53	114.50 ± 23.47	10.09%	< 0.001	0.59	−0.57
Physical Comfort	50	41.80 ± 7.63	38.17 ± 9.35	8.68%	< 0.001	0.32	−0.34
Emotional State	40	31.90 ± 6.71	32.51 ± 6.72	1.91%	0.247	0.11	0.08
Psychological Support	20	19.06 ± 1.94	18.28 ± 2.95	4.09%	0.013	0.23	−0.22
Physical Independence	20	17.63 ± 3.96	11.70 ± 6.31	33.63%	< 0.001	0.68	−0.87
Pain	20	17.11 ± 4.23	14.54 ± 4.78	15.02%	< 0.001	0.47	−0.46
